# Supplementary material for: Real-time environmental surveillance of SARS-CoV-2 aerosols
Source: Nat Commun. 2023 Jul 10;14:3692. doi: 10.1038/s41467-023-39419-z (PMC10333287; doi:10.1038/s41467-023-39419-z)
Supplement: Supplementary file 1 — Supplementary Information [file 41467_2023_39419_MOESM1_ESM.pdf]

**Supplementary Information for**

# Real-time environmental surveillance of SARS-CoV-2 aerosols

Joseph V. Puthussery[1], Dishit P. Ghumra[1], Kevin R. McBrearty[2], Brookelyn M. Doherty[2], Benjamin J. Sumlin[1], Amirhossein Sarabandi[1], Anushka Garg Mandal[1,3], Nishit J. Shetty[1a], Woodrow D. Gardiner[2], Jordan P. Magrecki[2], David L. Brody[4,5], Thomas J. Esparza[4], Traci L. Bricker[6], Adrianus C.M. Boon[6,7], Carla M. Yuede[8*], John R. Cirrito[2*] and Rajan K. Chakrabarty[1*]

**Affiliations:**

[1]Center for Aerosol Science and Engineering, Department of Energy, Environmental and Chemical Engineering, Washington University in St. Louis, St. Louis, MO 63130, USA

[2]Department of Neurology, Hope Center for Neurological Disease, Knight Alzheimer's Disease Research Center, Washington University, St. Louis, MO 63110, USA.

[3]Department of Chemical Engineering, Indian Institute of Technology Bombay, Mumbai, 400076, India.

[4]National Institute of Neurological Disorders and Stroke, Bethesda, Maryland, United States of America.

[5]Department of Neurology, Uniformed Services University of the Health Sciences, Bethesda, Maryland, United States of America.

[6]Department of Medicine, Washington University, St. Louis, MO 63110, USA.

[7]Departments Molecular Microbiology, and Pathology and Immunology, Washington University School of Medicine, St. Louis, Missouri, USA.

[8]Department of Psychiatry, Washington University School of Medicine, Campus Box 8134, 660 South Euclid Ave., St. Louis, MO 63110, USA

[a]Now at: Civil and Environmental Engineering, Virginia Tech, Blacksburg, VA 24061, USA

*To whom correspondence may be addressed: chakrabarty@wustl.edu (R.K.C), cirritoj@wustl.edu (J.R.C), and yuedec@wustl.edu (C.M.Y)

**Supplementary Method 1: Wet Cyclone Design and Computation Fluid Dynamics (CFD) Simulations**

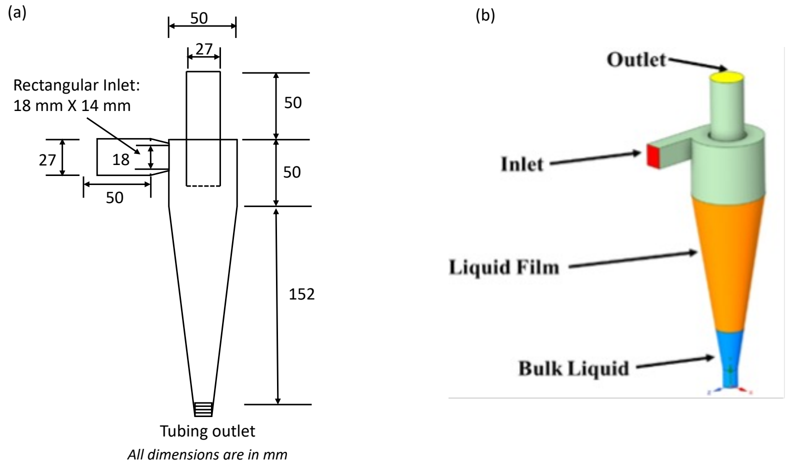


**Supplementary Fig. 1 Cyclone characterization.** (a) dimensions of the glass cyclone, and (b) CFD boundary conditions.

Supplementary Fig.1a shows the dimensions of the wet cyclone used in this study. The cyclone was custom-designed and built using glass (Adams & Chittenden Scientific Glass Coop, CA, USA).

CFD Simulations: In a high-flow rate cyclone, the particle separation depends on the swirling flow pattern of the incoming particles. The swirling airflow forces the particles to impact the cyclone wall, which drives particle separation. The flow regime inside a cyclone is highly turbulent and anisotropic. The carrier phase is the gas (ambient air), and the dispersed phase is the particle (water-liquid). Ansys Fluent 2021 R1 was used for the simulations, and the Reynold stress model was applied for modeling the flow field in the cyclone body.[1]

The solution algorithm used for solving the pressure-linked equations is SIMPLE, which is a semi-implicit method. The spatial discretization applied is as follows: PRESTO! discretization for pressure, QUICK method for momentum equation, second order upwind for turbulent equations, and first order for Reynold stress terms. ICEM-CFD was used to create a structured hexahedral mesh of the cyclone design.

Boundary Conditions

Supplementary Fig.1b shows the computational domain of the simulated wet cyclone. The volumetric flow rate at the cyclone inlet was set as 1000 LPM (equivalent to 66.138 m/s inlet velocity). The boundary conditions assumed for this simulation are summarized in Supplementary Table 1.

As shown in Supplementary Fig.1b, we assumed a thin liquid film forms along the walls of the cyclone. The bottom portion of the wet cyclone (50 mm) was assumed to be filled with liquid (based on experimental observation). Particles that came in contact with cyclone cone walls (orange portion) and the bottom liquid surface are assumed trapped/collected. Additionally, we assumed that particles impacting the top cylindrical section of the cyclone were not collected. In other words, it is assumed that particles coming in contact with the cyclone wall (conical section) or liquid surface at the bottom of the cyclone are trapped. To make sure that there is an equal mass between the inlet and outlet, the average volumetric mass between the inlet and outlet was monitored during the simulations. Supplementary Fig. 2 shows the particle trapped inside the wet cyclone for sizes ranging from 0.1 to 5 um. The cut-off diameter where the cyclone efficiency = 50% is 0.4 um.

**Supplementary Table 1**. Boundary condition assumed for the CFD modeling

| **Boundary Condition** | **Values** |
|---|---|
| **Inlet volumetric flow rate** | 1000 LPM |
| **Inlet velocity** | 66.136 m/s |
| **Pressure at the inlet** | Atmospheric pressure (1 atm) |
| **Particle** | Water-liquid |
| **Particle density** | 997 $\frac{kg}{m^3}$. |
| **Wall entrapment** | The whole conical section and bottom of the cone part filled with bulk liquid |
| **Inlet temperature** | 25 $°C$ |
| **Number of particles injected at each time step** | 260 |
| **Time steps** | 0.0001 second |
| **Number of time step** | 20000 |

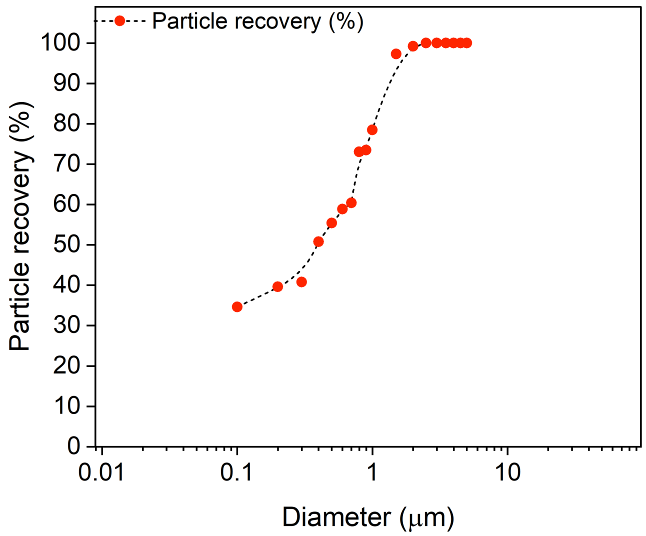


**Supplementary Fig. 2 Particle Recovery:** Size-specific particle recovery in the wet cyclone based on CFD modeling.

**Supplementary Method 2: Micro-immunoelectrode (MIE) biosensor**

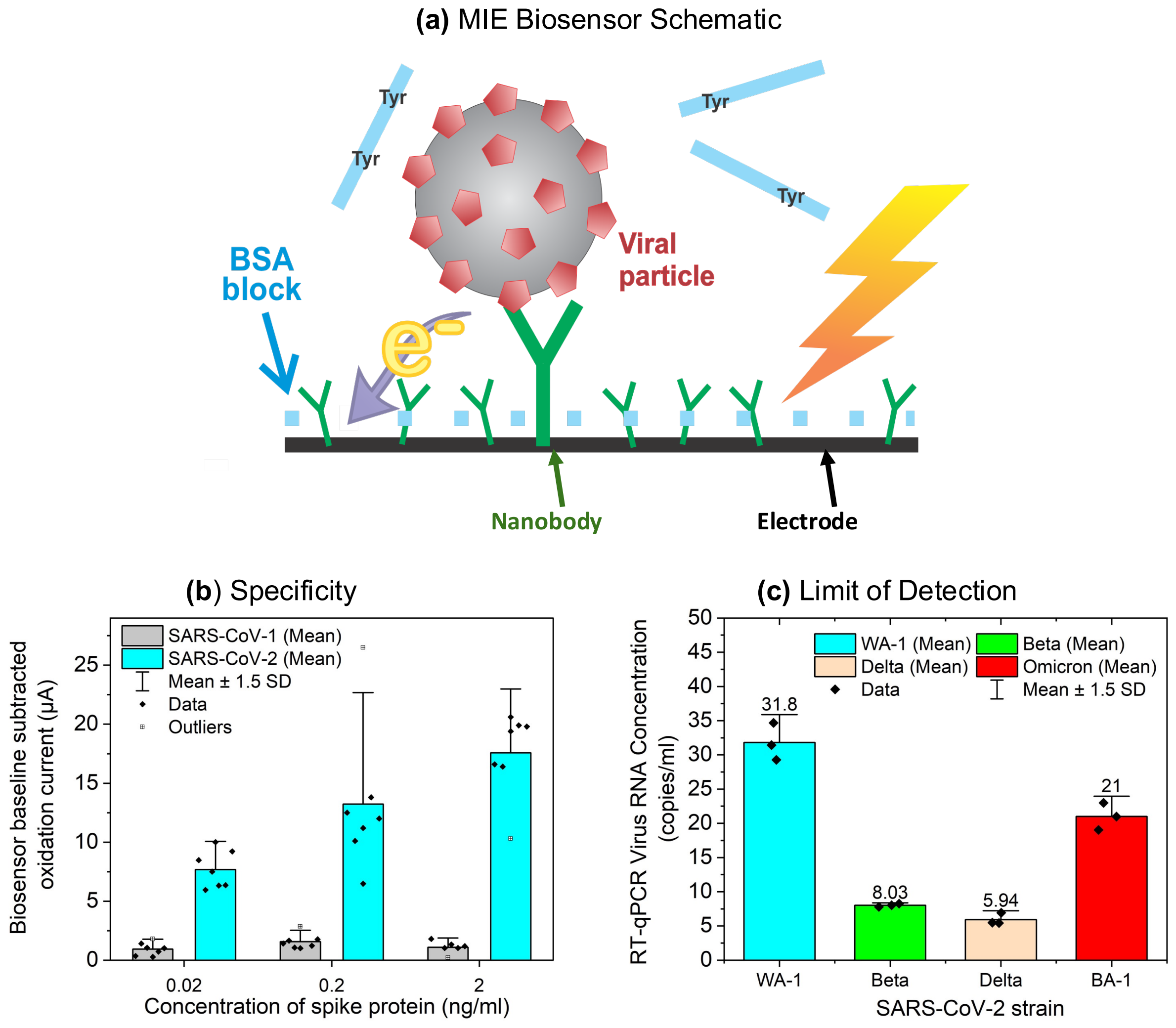


**Supplementary Fig. 3 MIE biosensor overview**: (a) Working principle, (b) specificity plot which shows the average biosensor signal for different concentrations of SARS-CoV-1 and SARS-CoV-2. The data are presented as mean ± 1.5 SD of n=7 independent experiments, and (c) sensitivity plot showing the lowest viral concentrations detected by the biosensor. The data are presented as mean ± 1.5 SD of n=3 independent experiments. Note, all biosensor measurements were repeated by scanning the same sample 5 times. All 5 scan gave consistent positive or negative reading.

The MIE biosensor uses screen-printed carbon electrodes (SPiCE, Catalog# SP-1401, BASi Research Products, West Lafayette, IN). A nanobody derived from llamas, which selectively binds to SARS-CoV-2 spike protein, is covalently attached to the electrode surface (Supplementary Fig. 3a). The method for detecting virus aerosols is based on the MIE technique developed in the Cirrito Laboratory.[2,3] The SARS-CoV-2 detection works based on the electroactivity of Tyrosine amino

acids present on the viral particle that can be oxidized on the carbon electrode. The SPiCEs are pre-treated using phosphate-buffered saline (PBS), and the surface is pre-blocked in a solution of 1% bovine serum albumin (BSA) to avoid binding of non-specific electroactive species. The MIE biosensor uses square wave voltammetry (SWV) to measure the oxidation of tyrosine amino acid present in the viral particle. When SWV is performed, tyrosine oxidation at ~0.65 V releases electrons which the sensor detects as current. The height of the peak oxidation current is proportional to the concentration of SARS-CoV-2 virions attached to the electrode surface.

The llama-derived nanobody used in this electrode has a moderate affinity to the SARS-COV-2 spike (S) protein to allow for dissociation. This ensures that the nanobody binding capacity of the MIE remains open to accept new SARS-CoV-2 spike (S) protein and thereby prolong the longevity of the MIE biosensor. Based on lab characterization, each MIE can be used for ~70 sample scans, which equates to ~ 35 real-time sample measurements (i.e., 35 baseline scans+35 sample scans).

We measured biosensor oxidation current at different concentrations of SARS-CoV-1 and SARS-CoV-2 spike proteins. SARS-CoV-1 and SARS-CoV-2 spike proteins have 70% similarity in their genetic makeup. The significantly higher oxidation current measured by the MIE biosensor for SARS-CoV-2 spike proteins (Supplementary Fig. 3b) compared to SARS-CoV-2 spike proteins at concentrations ranging from 0.02 to 2 ng/ml highlights the excellent specificity of the MIE biosensor used in this study.

The MIE biosensor LoD was calculated by sequentially diluting a pure stock solution of the inactivated virus, and the oxidation current ($I_{ox}$) was measured based on SWV. The lowest viral concentrations detected by the biosensor for the USA/WAa1/2020 (WA1), Beta (B.1.351), Delta (B.1.617.2), and Omicron (BA.1) strains of SARS-CoV-2 are shown in Supplementary Fig. 3c. The LoD for the different strains varied from 6-32 RNA copies/ml. The low LoD obtained shows the ultrasensitive SARS-CoV-2 virus detection capability of the MIE biosensor.

***Electrode preparation***

To enable efficient binding of the llama-derived nanobody on the electrode surface and enhanced tyrosine oxidation, the SPCEs are pretreated in PBS using a triangular waveform from 0 to 3V at 70 Hz for 20 seconds, followed by holding at -0.8 V and 1.5 V for 5 sec and 10 sec respectively. The activation of carboxylic groups on the electrode surface was carried out by using 0.4 M EDC ((N-(3-Dimethylaminopropyl)-N'-ethylcarbodiimide hydrochloride) and 0.1 M NHS (N-Hydroxysuccinimide) solution (Thermo Scientific, IL, USA) to form a semi-stable reactive amine NHS ester. To attach the nanobody to the electrode surface, the activated electrodes are placed in a solution of the nanobody and incubated for a period of 10 min at room temperature, followed by 4$^{o}$C overnight. Finally, the biosensors are incubated with 0.05% ethanolamine to inactivate the reactive amine sites and with 1% BSA solution to block non-specific protein binding sites.

**Supplementary Method 3: pAQ monitor operation**

A) **<u>pAQ device initialization (duration 5 minutes)</u>**

1. The peristaltic pump sends 15 ml buffer solution to the wet cyclone, and the wet cyclone begins sampling ambient air at 1000 lpm.
2. 2 ml of calibrant is pumped into the MIE vial housing the biosensor. Biosensor baseline reading is recorded by performing SVW. The MIE vial is emptied and ready for the next sample.

B) **<u>pAQ monitors continuous sample collection</u>**

1. **Time: 0-15 s**: At the end of the 5-min sample collection, 2 ml of the sample solution is transferred from the wet cyclone to the submerged MIE vial.
2. **Time: 30 s – 1 min:** The remaining liquid inside the wet cyclone is emptied into the waste, and the wet cyclone is filled with fresh buffer and begins the next round of sample collection.
3. **Time: 1 min – 2 min 30 s:** SWV is performed on the sample in the MIE vial to measure the peak oxidation current at ~0.65 V. This peak oxidation current is subtracted from the previously measured biosensor baseline reading. This baseline subtracted peak oxidation current is normalized with a virus-free control oxidation current (explained later). We assume the sample to be positive if the peak oxidation current after normalizing to control is greater than 1.15 (i.e., > blank + 3SD).
4. **Time: 1 min – 4 min 45 s:** The MIE vial is emptied, and 2 ml of calibrant is transferred to the submerged MIE vial housing the biosensor. Biosensor baseline reading is recorded by performing SVW. Frequent baseline measurements before every sample measurement ensure that any instrument drift-related errors are minimized. The user may examine the stability and repeatability of the baseline measurements for post-measurement quality checks.
5. **Time: 4 min 45 s to 5 min:** The MIE vial is emptied and ready for the next sample
6. Repeat steps B1 to B4 for a user-defined number of loops.
7. We have an optional wet cyclone decontamination step where the pump injects 15 ml hypochlorous acid (HOCl) into the wet cyclone (~1 minute 30 s) and then rinses it with buffer two times (~ 3 minutes). This can be programmed to happen after every 10 sample collection runs (this frequency can be varied). We expect this periodic decontamination of the wet cyclone would be needed when running the pAQ monitor continuously for several days in an indoor environment with high concentrations of diverse airborne pathogens. *Note the lab experiment results reported in this study were*

*not performed in continuous mode. The wet cyclone was manually decontaminated, cleaned, and dried after every sampling cycle.*

### C) pAQ virus-free control air measurement (duration 5 min)

Before any sampling, a 5-min virus-free air control measurement must be stored in the device. This control would preferably be taken from the indoor environment with similar aerosol composition as the study area. All sample measurements are reported after normalizing to this control. We recommend taking control air measurements whenever moving the device to a new location. For our lab experiments, we sampled aerosolized PBS using the wet cyclone, which was used as a control for sample measurement normalization.

**Supplementary Method 4: Cells and viruses**

Vero cells expressing human ACE2 and TMPRSS2 (Vero-hACE2-hTMPRSS2, gift from Adrian Creanga and Barney Graham, NIH)[4,5] were cultured at 37°C in Dulbecco's Modified Eagle medium (DMEM) and are supplemented with 10% fetal bovine serum (FBS), 10 mM HEPES (pH 7.3), 100 U/mL of Penicillin-Streptomycin, and 10 µg/mL of puromycin. Vero cells expressing TMPRSS2 (Vero-hTMPRSS2)[5] were cultured at 37°C in Dulbecco's Modified Eagle medium (DMEM) supplemented with 10% fetal bovine serum (FBS), 10 mM HEPES (pH 7.3), 100 U/mL of Penicillin-Streptomycin, and 5 µg/mL of blasticidin.

The original strain of SARS-CoV-2 (strain 2019-nCoV/USA-WA1/2020), and the Delta (B.1.617.2) and Omicron (B.1.1.529) variant of SARS-CoV-2 were propagated on Vero-hTMPRSS2 cells. Plaque assay on Vero-hACE2-hTMPRSS2 cells was used to determine the infectious virus titer.

A culture supernatant containing an infectious virus was treated for 18 hours with 1:1000 dilution of beta-Propiolactone (BPL) for the inactivation of SARS-CoV-2. After the inactivation by BPL at 37°C for a period of one hour, the inactivation of SARS-CoV-2 was confirmed by plaque assay on Vero-hACE2-hTMPRSS2 cells as reported previously. The assay also includes an inactivated sample and positive control.[6]

**Supplementary Method 5: Chamber Experiment Set Up**

Supplementary Fig. 4 shows the basic layout of the 21 $m^3$ chamber experiment setup. 100 ul aliquots of the stock virus were thawed and diluted in PBS to desired concentrations. The Collison nebulizer (CH Technologies, USA) filled with inactivated WA-1 (diluted in PBS) was placed in one corner of the chamber. The nebulizer outlet was at a height of ~3 feet from ground level. Two fans were placed on opposite corners of the chamber to achieve uniform mixing inside the chamber (fan height = 1 -1.5 feet). The wet cyclone and the BioSampler® were placed inside the chamber, approximately at the center, but sufficiently far apart to not interfere with either of their sampling collection (chamber floor area: 7.13$m^2$ or 11'X7"). The stainless-steel test chamber has multiple welded stainless-steel ports (1/4" diameter) that can be connected to external air sampling devices for chamber air sampling. We connected one of these sampling ports to the LSS and another to the Scanning Mobility Particle Sizer (SMPS). We ensured that all the sampler inlets were at similar heights so that all the instruments sampled air at the same height (4 to 4.5 feet from the floor). Note that the SKC BioSampler® and LSS used in this experiment are 2 of the most widely used bioaerosol samplers because of their high bioaerosol collection efficiency. For instance, Eiguren et al.[7] report the particle collection efficiency of the LSS to be greater >95% for particles in the size range of 10 nm to 2500 nm. Similarly, Li et al.[8] reported a U-shaped physical particle collection of the SKC BioSampler® with >70% recovery for particles larger than 300nm and less than 10 nm in diameter.

**Supplementary Table 2.** Summary of the instruments used in the chamber experiments.

| **Instrument** | **Manufacturer** | **Flow rate (LPM)** | **Collection Media** |
|---|---|---|---|
| Wet Cyclone | Assembled and built at Washington University in St. Louis, MO, USA<br><br>Glass blowing work: Adams & Chittenden Scientific Glass Coop, California, USA. | 1000 | PBS (~30 ml) |
| BioSampler® | SKC Inc., PA, USA | 12.5 | PBS (5 ml) |
| Liquid Spot Sampler™ (LSS) | Aerosol Devices, CO, USA | 1.5 | PBS (0.3 ml) |
| Scanning Mobility Particle Sizer (SMPS) | TSI, MN, USA | 1.5 | Not applicable |

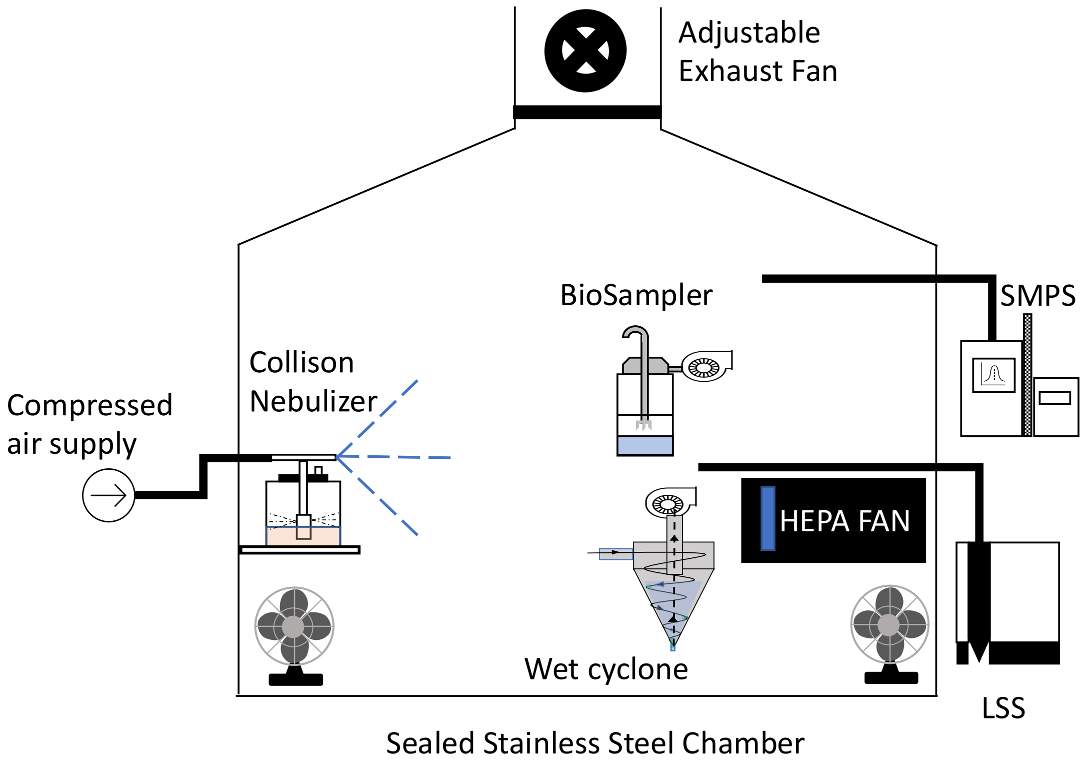


**Supplementary Fig. 4 Chamber Experiments:** The layout of the chamber experiment set-up to test the virus collection performance of the wet cyclone.

Experiment procedure:

1. Chamber air cleaning: The exhaust fan shutter is completely opened to facilitate maximum ventilation. The vertical HEPA fan filter (80 CFM, 99.97% particle removal; Dayton Model: 2HPC3A) is switched on to filter out all particles suspended inside the chamber. The chamber is cleaned for 60 minutes before the start of any experiment. The size distribution and particle number concentration (PNC) inside the chamber are continuously monitored using the SMPS.

2. The Collison nebulizer filled with the stock virus was then connected to a 20-psi compressed particle-free air supply. The nebulizer was left running for 5 minutes to obtain stable aerosol generation. The typical particle size distribution measured inside the chamber is shown in Supplementary Fig. 5.

3. After 5 minutes, all three samplers were switched on, and air sampling was performed for 10 minutes.

4. The three samplers and the Collison nebulizer were turned off after 10 minutes of sample collection. Step 1 was repeated, and the PNC was monitored using the SMPS.

5. When PNC reached its original background concentration, the door to the chamber was opened, and the samples were collected and stored for RT-qPCR analysis inside a

refrigerator at 4°C. All RT-qPCR PCR analyses were completed within 18h of sample collection.

6. After retrieving the samples, the wet cyclone, the BioSampler®, and LSS sample vials were washed multiple times and decontaminated using 70% ethanol. All the samplers were then rinsed with milli-q water and air-dried before the next round of sample collection.

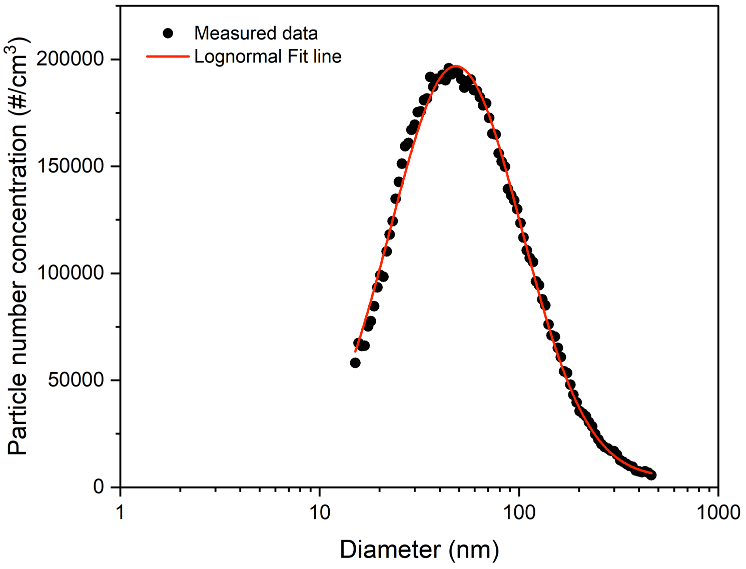


**Supplementary Fig. 5 Particle Size Distribution:** Typical probability density function of aerosol mobility size measured inside the test chamber during the experiments. This distribution is representative of the SARS-CoV-2 aerosol size distribution found in an indoor environment.

**Supplementary Method 6. Sample collection from apartments with SARS-CoV-2 positive patients**

We collected indoor air samples using the wet cyclone sampler from the self-isolating bedrooms of two SARS-CoV-2 positive individuals. The wet cyclone assembly was dropped off late evening in front of the two volunteers' apartments. The volunteers were provided with a sampling kit that consisted of 15 ml PBS solution vials (4 nos), an insulated ice box with gel ice packs to store the samples, clean sterilized 15 ml centrifuge tubes (4 nos) to store the collected samples, and 10% bleach solution. The volunteers were instructed on how to perform the sample collection virtually over a video call.

In both apartments, the wet cyclone sampler was placed on top of a nightstand next to the volunteer's bed (~1 foot away from the bed). The wet cyclone sampled air at the height of ~3.5 feet from ground level. The exact distance between the sampler and the patients was not measured (due to logistical and safety constraints), but we instructed the volunteers not to exhale directly into the cyclone inlet. When collecting the sample, the volunteers were asked to remain inside the bedroom but stay slightly away (at least 2 feet) from the wet cyclone inlet.

After 5 minutes of sample collection, the volunteers took out the samples using the manual detachable 20 ml syringe screwed onto the wet cyclone. The samples were immediately stored inside the ice box. After collecting all the samples, the volunteers rinsed the wet cyclone using the 10% bleach solution and rinsed it multiple times with tap water. The volunteers then moved all the collected samples, wet cyclone, vacuum pump, and sampling kit to the front door outside their apartments. The researchers again surface disinfected all the sampling apparatus by spraying 70% ethanol and wiping with disinfectant wipes outside the apartment. The entire sampling apparatus and samples were placed in a secondary plastic storage bag/container before transporting to the lab. All the samples were analyzed by RT-qPCR on the same day of sample pickup. The RT-qPCR results from apartment samples were compared with those from a clean, unoccupied, well, ventilated conference hall, which we assumed as a control air sample.

**Supplementary Table 3.** Summary of the apartment air sampling study

| | Volunteer 1 | Volunteer 2 |
|---|---|---|
| Residence description | • Volunteer 1 self-isolated in an 11'X15' bedroom of 1 bed-hall-kitchen (BHK)+1 bath apartment (entire apartment area 531 square feet)<br>• The apartment had central air conditioning. The volunteer self-reported having switched set the indoor temperature as 24 °C. No other indoor air quality measurements were recorded. | • Volunteer 2 self-isolated in a 10'X17''bedroom of 1 bed-hall-kitchen (BHK)+1 bath apartment (entire apartment area 750 square feet)<br>• The apartment had central air conditioning. The volunteer self-reported having set the indoor temperature as 23°C. No other indoor air quality measurements were recorded. |
| Case description | • Volunteer 1 first tested positive for SARS-CoV-2 on Sep 3, 2022 (based on self at home antigen test) and then tested positive again on Sep 7, 2022 (based on a nasal swab RT-PCR test).<br>• The volunteer self-reported 2 concurrent negative nasal swabs based on home antigen tests on Sep 11, 2022, and Sep 12, 2022.<br>• The volunteer reported experiencing the following symptoms from Sep 3 – 5, 2022: fever, chills, fatigue, loss of appetite, and cough. The volunteer did not show any symptoms after Sep 6, 2022. | • Volunteer 2 first tested positive for SARS-CoV-2 on Sep 4, 2022 (based on self at home antigen test) and then tested positive again on Sep 7, 2022 (based on self at home antigen test).<br>• The volunteer self-reported two concurrent negative nasal swabs based on home antigen tests on Sep 12 and Sep 13, 2022.<br>• The volunteer reported experiencing the following symptoms from Sep 4 – 8, 2022: fever, fatigue, and cough. The volunteer did not show any symptoms after Sep 10, 2022. |
| Vaccination status | • Fully vaccinated<br>• First Dose + Booster shot | • Fully vaccinated<br>• First Dose + Booster shot |

**Supplementary Table 3.** Continued…

| | Volunteer 1 | Volunteer 2 |
|---|---|---|
| Dominant SARS-CoV-strain[9] | BA.5 | BA.5 |
| Sample collection | • Indoor air samples were collected on Sep 6 and Sep 7, which was ~4 and 5 days from the time they first tested positive. Volunteer was asymptotic during the sample collection period.<br>○ Sample 1: Sep 6, 2022, 10 PM<br>○ Sample 2: Sep 6, 2022, 11 PM<br>○ Sample 3: Sep 7, 2022, 8 AM<br>○ Sample 4: Sep 7, 2022, 9 AM | • Indoor air samples were collected on Sep 10 and Sep 11, which was ~6 and 7 days from the time they first tested positive. Volunteer reported mild cough during the sample collection period and reported no other symptoms.<br>○ Sample 1: Sep 10, 2022, 10 PM<br>○ Sample 2: Sep 10, 2022, 11 PM<br>○ Sample 3: Sep 11, 2022, 8 AM |

## Supplementary Method 7: pAQ Monitor Sensitivity Experiments

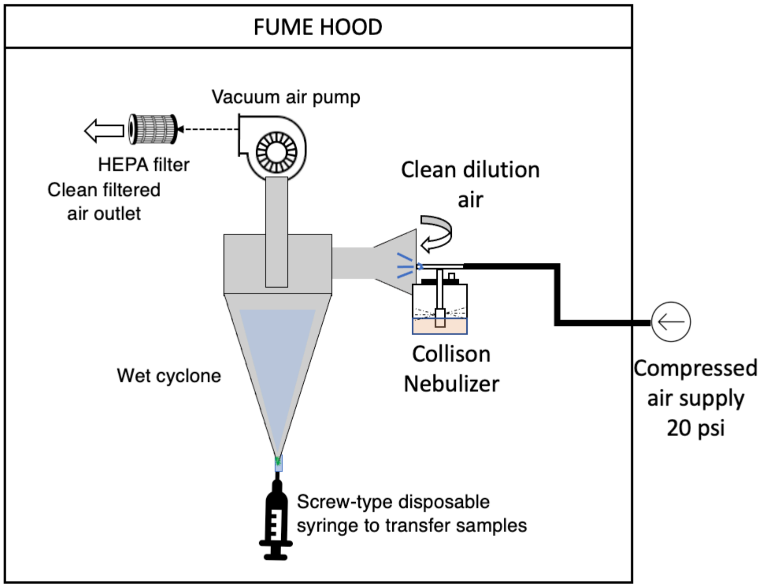


**Supplementary Fig. 6 pAQ Monitor Sensitivity Experiments**: The virus aerosolization experiment set-up to determine the pAQ sensitivity.

Different concentrations of test SARS-CoV-2 were prepared by diluting inactivated SARS-CoV-2 (WA-1 and BA-1) stock in PBS solution. The test solution was aerosolized using a Collison Nebulizer inside a fume hood. The Collison aerosol generation port was placed directly in the center of the wet cyclone inlet, using custom built wide mouth glass conical adaptor (~4" diameter opening)**.** Here, we assume the strong suction at the inlet of the wet cyclone will ensure that all the aerosols generated by the Collison nebulizer enter the cyclone. Along with the virus aerosols, ~993 L of clean lab air also enters the cyclone. We expect substantial volumetric dilution and drying of the aerosols when it enters the cyclone. Average lab temperature = 70 °F and indoor RH = 14% – 20%. The low RH conditions in our lab could lead to rapid evaporation[10] of the aerosols generated by the Collison nebulizer and shrink the aerosol before it enters the wet cyclone. This, in turn, would lower the overall particle collection efficiency of the wet cyclone, as shown in Supplementary Fig.2. The device sensitivity reported in this study can be assumed as conservative estimates assuming extreme RH conditions.

The wet cyclone was filled with 15 ml PBS solution, and 5 min aerosolized virus sampling was performed. After 5 min, the sample was taken out manually using a disposable syringe screwed onto the base of the wet cyclone. The samples collected were manually divided into two portions, one was analyzed using the biosensor, and the other was analyzed by RT-qPCR (within 18h of sample collection). After retrieving the samples, the wet cyclone was decontaminated using

70% ethanol. The wet cyclone was washed, rinsed with milli-q water, and air-dried before the next round of sample collection.

Note that the pAQ monitor sensitivity data for WA-1 was obtained by compiling results from 3 separate experiments performed on different days (number of samples analyzed = 4, 4, and 5 on days 1, 2, and 3, respectively), using different MIE electrodes and different aerosolized virus concentrations. This ensures the sensitivity reported incorporates errors associated with MIE biosensor biases, any inter-day measurement/sampling biases, and biases associated with aerosolized virus concentration. We also measured RNA concentration in the wet cyclone collected aerosolized WA-1 samples using RT-qPCR and compared it to the biosensor oxidation current. The biosensor oxidation signal, in general, increased with an increase in RNA concentration measured using the RT-qPCR (Supplementary Fig. 7). Due to the limited availability of the pure inactivated BA-1 strain, all the BA-1 aerosolization experiments were completed on the same day using the same electrode but at different aerosolized virus concentrations.

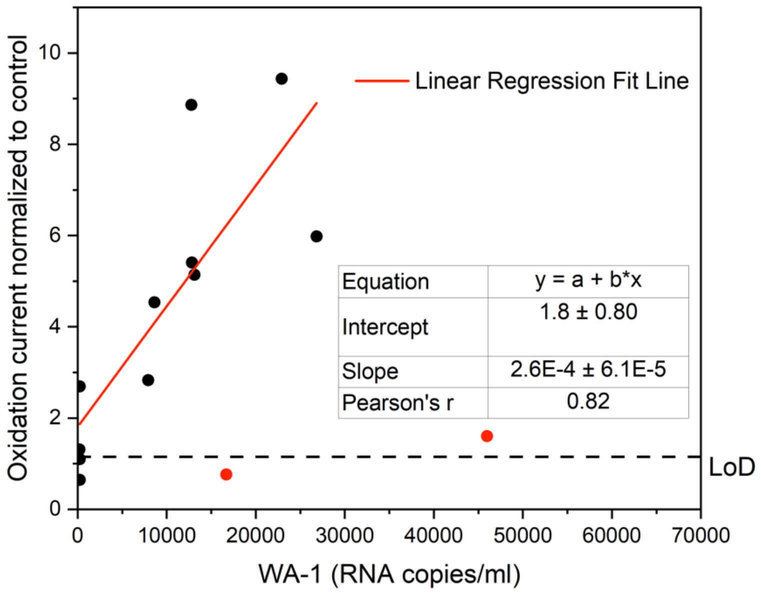


**Supplementary Fig. 7 pAQ Monitor Sensitivity**: Biosensor oxidation current normalized to control vs. WA-1 RNA concentration determined using RT-qPCR. The dashed line indicates the biosensor LoD. Red circles indicate data points marked as outliers and removed prior to the regression analysis.

**Supplementary Method 8: RT-qPCR**

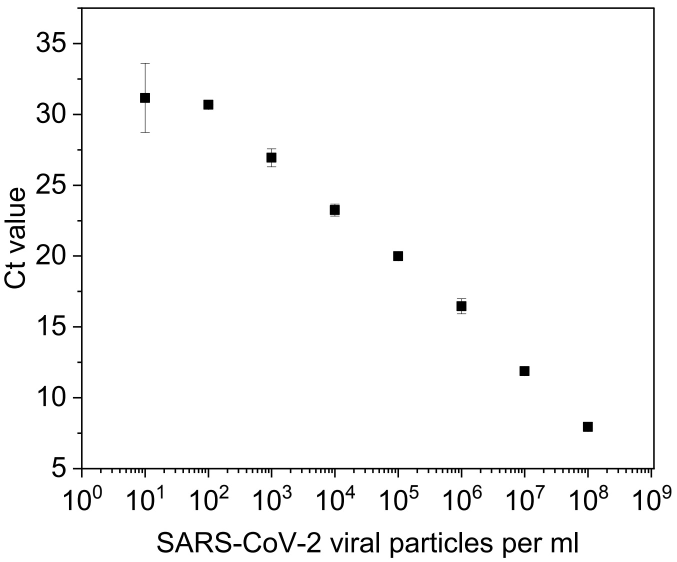


**Supplementary Fig. 8 RT-qPCR Standard Curve.** A fresh calibration curve was prepared prior to any RT-qPCR experiments to convert the sample Ct values to RNA concentration in copies/ml. Each data point shows the average Ct value (± standard error of mean) of n=3 technical replicates of the dilution series.

We use the following criteria while reporting the SARS-CoV-2 RNA concentration:

Ct> 36: SARS-CoV-2 not detected.

Ct > 30 and Ct < 36: SARS-CoV-2 positive. These samples can be considered to be weakly positive samples with very low RNA concentrations that cannot be accurately quantified based on our protocol.

Ct <30: SARS-CoV-2 positive, and we report the concentration after converting the units to RNA copies/$m^3$ based on the calibration curve shown in Supplementary Figure 8.

The steps for converting Ct counts to RNA copies/$m^3$ are as follows:

- The Ct value of the virus aerosol samples collected inside the wet cyclone was determined.
- This Ct value was then converted to RNA copies/ml using the calibration curve similar to that shown in Supplementary Fig.8.
- Finally, the concentration in the liquid solution was converted to the volume of air normalized concentration using equation 1 shown below:

$$\text{pAQ Monitor RNA concentration}\left(\frac{\text{RNA copies}}{\text{m}^3\text{ of air}}\right)=\frac{\left[\begin{matrix}\text{RNA concentration in}\\\text{collected liquid (copies/ml)}\end{matrix}\right]*\left[\begin{matrix}\text{volume of liquid inside}\\\text{the wet cyclone(ml)}\end{matrix}\right]}{\text{Volume of air sampled }\left(\text{m}^3\right)} \quad \ldots \text{ eq (1)}$$

The volume of liquid inside the cyclone at the end of sampling was measured manually. The volume of air sampled was calculated by multiplying the sampling flow rate [(~1000 liters per minute (lpm)] with the sampling duration (5 or 10 min).

**Supplementary Discussion 1: pAQ Monitor Cost Analysis**

Fixed cost for building a single pAQ Monitor:

- The cost of building a single wet cyclone air sampler, which includes the cost of tubing connectors, labor, design, and shipping = $600 - $700
- The cost of building the MIE measurement unit that comprises the biosensor, automated liquid handling pumps, microcontroller, potentiostat measurement setup, and miscellaneous accessories = $700 - $1000
- Cost of the air blower/vacuum air pump = $100 - $200
- The total cost to build and design a single pAQ unit in a research laboratory will range from $1400 - $1900. We are currently working with an experienced industry manufacturer for scale-up and mass production. This collaboration will likely bring down the cost significantly.

Operation cost in the laboratory:

- Cost of PBS buffer (~20 ml is consumed per sample run) = ~US$ 0.08 per sample
- Cost of calibrant (~3 ml is consumed per sample run) = ~ US$ 0.18 per sample
- Cost of decontaminant (HOCl; ~20 ml is consumed per sample run) = ~ US$ 0.04 per sample
- The cost of the MIE biosensor = US$ 0.1 per sample
- Total operation cost in laboratory = US$ 0.40 per sample

The MIE biosensor used in the study can be screen printed using a laboratory scale screen printer or industrial screen printers. Therefore, the final cost of the biosensor would depend on the production scale. Moreover, the nanobody used in this electrode has a moderate affinity to the SARS-CoV-2 spike (S) protein to allow for dissociation. This ensures that the nanobody binding capacity of the MIE remains open to accept new SARS-CoV-2 spike (S) protein and thereby prolong the longevity of the MIE biosensor. Based on lab characterization, each MIE can be used for ~70 sample scans. This reusability of the biosensor helps us reduce the cost per sample.

Scaling up the production will significantly reduce the operation and fixed cost of the pAQ monitor. For example, operating a single pAQ monitor in longitudinal virus monitoring mode for a 30-day period and 12 air samples analyzed per hour will work out to ~8700 samples analyzed per month. Such longitudinal virus sampling using a single pAQ will require ~250 electrodes, ~174 L of buffer, and ~26 L of calibrant per month. HOCl consumption is variable and will be based on the user-defined decontamination frequency (~ 3 L a month). If more than 100,000 such units run in parallel at different locations, the monthly demand for the consumable (electrode, calibrant, and buffer) will also be high. If the electrodes are manufactured in bulk using industry-scale screen printers (> 150,000 electrodes a day),[11] the cost of the MIE biosensor will go down to <US$ 0.006 per sample analyzed. Furthermore, the MIE biosensor design can be optimized to help improve the electrode working time. We anticipate the operation life of a single electrode will increase from 70 scans to over 100 scans per electrode after optimization, bringing down the operation cost even lower. Similarly, the cost of the buffer, calibrant, and decontaminant will also go down by 10 folds when purchased in bulk. Overall, based on the production scale, the cost per sample analyzed can be a few cents or even lower.

## References


1. Song, C. *et al.* Numerical analysis of forces exerted on particles in cyclone separators. *Powder Technol.* **294**, 437–448 (2016).
2. Yuede, C. M. *et al.* Rapid in vivo measurement of ß-amyloid reveals biphasic clearance kinetics in an Alzheimer's mouse model. *J. Exp. Med.* **213**, 677–685 (2016).
3. Prabhulkar, S., Piatyszek, R., Cirrito, J. R., Wu, Z. Z. & Li, C. Z. Microbiosensor for Alzheimer's disease diagnostics: Detection of amyloid beta biomarkers. *J. Neurochem.* **122**, 374–381 (2012).
4. Chen, R. E. *et al.* Resistance of SARS-CoV-2 variants to neutralization by monoclonal and serum-derived polyclonal antibodies. *Nat. Med.* **27**, 717–726 (2021).
5. Zang, R. *et al.* TMPRSS2 and TMPRSS4 promote SARS-CoV-2 infection of human small intestinal enterocytes. *Sci. Immunol.* **5**, eabc3582 (2020).
6. Darling, T. L. *et al.* mRNA-1273 and Ad26.COV2.S vaccines protect against the B.1.621 variant of SARS-CoV-2. *Med* **3**, 309-324.e6 (2022).
7. Eiguren Fernandez, A., Lewis, G. S. & Hering, S. V. Design and laboratory evaluation of a sequential spot sampler for time-resolved measurement of airborne particle composition. *Aerosol Sci. Technol.* **48**, 655–663 (2014).
8. Li, J. *et al.* Comparing the performance of 3 bioaerosol samplers for influenza virus. *J. Aerosol Sci.* **115**, 133–145 (2018).
9. CDC. COVID Data Tracker. *Centers for Disease Control and Prevention* 7–11 https://covid.cdc.gov/covid-data-tracker/#vaccinations-pregnant-women%0Ahttps://covid.cdc.gov/covid-data-tracker/#datatracker-home%0Ahttps://covid.cdc.gov/covid-data-tracker/#cases_casesper100k (2022).
10. Vejerano, E. P. & Marr, L. C. Physico-chemical characteristics of evaporating respiratory fluid droplets. *J. R. Soc. Interface* **15**, 20170939 (2018).
11. Torres, M. D. T., de Araujo, W. R., de Lima, L. F., Ferreira, A. L. & de la Fuente-Nunez, C. Low-cost biosensor for rapid detection of SARS-CoV-2 at the point of care. *Matter* **4**, 2403–2416 (2021).
