# Supplementary material for: Real-time environmental surveillance of SARS-CoV-2 aerosols
Source: Nat Commun. 2023 Jul 10;14:3692. doi: 10.1038/s41467-023-39419-z (PMC10333287; doi:10.1038/s41467-023-39419-z)
Supplement: Supplementary file 3 — Reporting Summary [file 41467_2023_39419_MOESM3_ESM.pdf]

nature portfolio

Corresponding author(s): Rajan K. Chakrabarty, John R. Cirrito and Carla M. Yuede

Last updated by author(s): Jun 2, 2023

Reporting Summary

Nature Portfolio wishes to improve the reproducibility of the work that we publish. This form provides structure for consistency and transparency in reporting. For further information on Nature Portfolio policies, see our Editorial Policies and the Editorial Policy Checklist.

Statistics

For all statistical analyses, confirm that the following items are present in the figure legend, table legend, main text, or Methods section.

n/a	Confirmed	
☐	☒	The exact sample size (*n*) for each experimental group/condition, given as a discrete number and unit of measurement
☐	☒	A statement on whether measurements were taken from distinct samples or whether the same sample was measured repeatedly
☒	☐	The statistical test(s) used AND whether they are one- or two-sided<br>*Only common tests should be described solely by name; describe more complex techniques in the Methods section.*
☒	☐	A description of all covariates tested
☒	☐	A description of any assumptions or corrections, such as tests of normality and adjustment for multiple comparisons
☐	☒	A full description of the statistical parameters including central tendency (e.g. means) or other basic estimates (e.g. regression coefficient) AND variation (e.g. standard deviation) or associated estimates of uncertainty (e.g. confidence intervals)
☒	☐	For null hypothesis testing, the test statistic (e.g. *F*, *t*, *r*) with confidence intervals, effect sizes, degrees of freedom and *P* value noted<br>*Give P values as exact values whenever suitable.*
☒	☐	For Bayesian analysis, information on the choice of priors and Markov chain Monte Carlo settings
☒	☐	For hierarchical and complex designs, identification of the appropriate level for tests and full reporting of outcomes
☐	☒	Estimates of effect sizes (e.g. Cohen's *d*, Pearson's *r*), indicating how they were calculated

Our web collection on statistics for biologists contains articles on many of the points above.

Software and code

Policy information about availability of computer code

Data collection	No software was used.
Data analysis	Basic descriptive statistics were performed using Origin Pro 2022 software. The computation fluid dynamic simulations were performed using Ansys Fluent 2021 R1 to determine the particle recovery inside the wet cyclone. Relative mRNA levels were calculated by the comparative Ct method using the ABI 12K Flex Software package version 1.3. SolidWorks (Dassault Systèmes, Vélizy-Villacoublay, France) software package was used to generate the 3D renderings of the pAQ monitor.

For manuscripts utilizing custom algorithms or software that are central to the research but not yet described in published literature, software must be made available to editors and reviewers. We strongly encourage code deposition in a community repository (e.g. GitHub). See the Nature Portfolio guidelines for submitting code & software for further information.

Data

Policy information about availability of data

All manuscripts must include a data availability statement. This statement should provide the following information, where applicable:

- Accession codes, unique identifiers, or web links for publicly available datasets
- A description of any restrictions on data availability
- For clinical datasets or third party data, please ensure that the statement adheres to our policy

The datasets generated during and/or analysed during the current study are provided in the source data file.

Human research participants

Policy information about studies involving human research participants and Sex and Gender in Research.

Reporting on sex and gender	Not relevant for our study
Population characteristics	Not relevant for our study
Recruitment	Not relevant for our study
Ethics oversight	Not relevant for our study. The Institutional Review Board deemed this project unnecessary for approval because: 1) the study did not collect information about a living human, 2) the study did not involve an interaction or intervention with a living human being performed for research purposes, 3) the study did not involve the collection or use of identifiable, private information, and 4) the study was not testing a device designed to diagnose or treat a medical condition. Informed consent was obtained from the two volunteers to collect and analyze indoor air samples from their apartments.

Note that full information on the approval of the study protocol must also be provided in the manuscript.

Field-specific reporting

Please select the one below that is the best fit for your research. If you are not sure, read the appropriate sections before making your selection.

☐ Life sciences ☐ Behavioural & social sciences ☒ Ecological, evolutionary & environmental sciences

For a reference copy of the document with all sections, see nature.com/documents/nr-reporting-summary-flat.pdf

Ecological, evolutionary & environmental sciences study design

All studies must disclose on these points even when the disclosure is negative.

Study description	This study describes the design,development, and performance evaluation of a proof-of-concept real-time pathogen air quality (pAQ) monitor to detect the presence of the SARS-CoV-2 virus in the air in under 5 minutes. The air sampler performance was evaluated using computer-aided numerical simulation, followed by testing in the lab by sampling aerosolized inactivated target virus inside a sealed chamber and/or fume hood to simulate real-world conditions. The results from the biosensor (which is a part of the pAQ monitor) were compared with measurements from a reference method (i.e., RT-qPCR) to determine the device sensitivity. The air sampler was also tested inside the apartments of 2 COVID positive patients to demonstrate its application for indoor virus surveillance
Research sample	Vero cells expressing human ACE2 and TMPRSS2 (Vero-hACE2-hTMPRSS2) were cultured at 37°C in Dulbecco's Modified Eagle medium (DMEM) and are supplemented with 10% fetal bovine serum (FBS), 10mM HEPES (pH 7.3), 100U/mL of Penicillin-Streptomycin, and 10 µg/mL of puromycin. Vero cells expressing TMPRSS2 (Vero-hTMPRSS2) were cultured at 37°C in Dulbecco's Modified Eagle medium (DMEM) supplemented with 10% fetal bovine serum (FBS), 10mM HEPES (pH 7.3), 100U/mL of Penicillin-Streptomycin, and 5 µg/mL of blasticidin.<br>The original strain of SARS-CoV-2 (strain 2019-nCoV/USA-WA1/2020), and the Delta (B.1.617.2) and Omicron (B.1.1.529) variant of SARS-CoV-2 were propagated on Vero-hTMPRSS2 cells. Plaque assay on Vero-hACE2-hTMPRSS2 cells was used to determine the infectious virus titer.<br>A culture supernatant containing an infectious virus was treated for 18 hours with 1:1000 dilution of beta-Propiolactone (BPL) for the inactivation of SARS-CoV-2. After the inactivation by BPL at 37°C for a period of one hour, the inactivation of SARS-CoV-2 was confirmed by plaque assay on Vero-hACE2-hTMPRSS2 cells as reported previously.<br>Inactivated SARS-CoV-2 viral particles were used to closely mimic actual viral particles, but in a non-infectious state. This sample type was the closest approximation of live virus that we could procure outside of a BSL3 facility.<br><br>Indoor environmental samples were collected from the apartments of 2 COVID positive patients.
Sampling strategy	Samples tested were lab-generated inactivated SARS-COV-2 aerosols. We aerosolized 3 different concentrations ("Concentration Level," "Replicate Runs"; Low, n = 3; Medium, n = 2; High, n = 4) of SARS-CoV-2 WA-1 strain and sampled the chamber air using the wet cyclone and 2 other commercial particles into liquid samplers. The number of replicate chamber experiments was dependent on the availability of the stock-inactivated virus. The low variability in the measured virus concentrations results from replicate runs demonstrated the repeatability of the experiment.<br>Additionally, the pAQ monitor sensitivity calculation, which incorporates the errors from the wet cyclone sample collection and biosensor detection step, was determined by nebulizing inactivated WA-1 (n = 13) and BA-1 (n=6) using a Collison nebulizer and sampling for 5 min using the wet cyclone. The number of replicate experiments depended on the availability of inactivated virus.<br>We also tested the virus sampling performance of the wet cyclone inside the apartments of 2 COVID positive patients.<br>For indoor air sampling, two COVID-positive volunteers collected 5 min air samples (n=3 and 4) from inside their bedrooms/ apartments. We chose a sample size of 3-4 indoor air samples in each apartment, to ensure we rule out any site-specific experimental bias or random error. No statistical methods were used to predetermine the sample size. The sample size allowed to

	keep the time from sample collection to sample analysis short (< 18 h) and consistent throughout the study
Data collection	The description of the apartment test conditions and dimensions were self-reported by the volunteers via email to one of the authors of this study.
Timing and spatial scale	For apartment indoor air sampling, Volunteer 1 collected four independent 5-minute indoor air samples Sample 1: Sep 6, 2022, 10 PM Sample 2: Sep 6, 2022, 11 PM Sample 3: Sep 7, 2022, 8 AM Sample 4: Sep 7, 2022, 9 AM<br>Volunteer 2 collected three independent 5-minute indoor air samples. Sample 1: Sep 10, 2022, 10 PM Sample 2: Sep 10, 2022, 11 PM Sample 3: Sep 11, 2022, 8 AM<br>The sampling time was chosen so as to cause least discomfort to the recuperating SARS-CoV-2 positive volunteers. The volunteers were instructed to wait for at least 1h between consecutive sample collection. Furthermore, to ensure consistency between the apartment and lab experiments, we ensured all virus samples collected were analyzed with 18h of sample collection. Considering all these factors, we requested the volunteers sample indoor air between 10 PM and 9 am.
Data exclusions	No data were excluded from the analysis
Reproducibility	All lab aerosolization experiments were repeated at least twice (typically > 3 times). All attempts at replications were successful, except a single sample collected using the LSS (Fig. 2 in the manuscript), where the instrument unexpectedly stopped in the middle of sample collection. This is noted in the source data file.<br>All biosensor-based virus measurements were repeated by scanning the same sample 5 times. All 5 scans gave a consistent positive or negative reading.
Randomization	For all RT-qPCR experiments the samples were randomly ordered prior to analysis. While analyzing the pAQ monitor collected aerosolized virus using the biosensor, no randomization was done. This is because, in our protocol (explained in the methods section and in Supplementary Method 3) the baseline biosensor reading needs to be always acquired prior to any sample measurements. Therefore, the samples and baseline calibration should always be done alternatively for the device to function properly.
Blinding	Complete blinding was not feasible as the researchers involved in lab experiments were also directly or indirectly involved in helping with conducting the lab sampling and apartment air sampling experiments.

Did the study involve field work? ☐ Yes ☒ No

Reporting for specific materials, systems and methods

We require information from authors about some types of materials, experimental systems and methods used in many studies. Here, indicate whether each material, system or method listed is relevant to your study. If you are not sure if a list item applies to your research, read the appropriate section before selecting a response.

Materials & experimental systems

n/a	Involved in the study
☒	☐ Antibodies
☐	☒ Eukaryotic cell lines
☒	☐ Palaeontology and archaeology
☒	☐ Animals and other organisms
☒	☐ Clinical data
☒	☐ Dual use research of concern

Methods

n/a	Involved in the study
☒	☐ ChIP-seq
☒	☐ Flow cytometry
☒	☐ MRI-based neuroimaging

Eukaryotic cell lines

Policy information about cell lines and Sex and Gender in Research

Cell line source(s)	Vero E6 cells (America Type Culture Collection (ATCC)-CRL1586)
Authentication	Cells were originally validated by the manufacturer. Cells were then sequenced to verify SARS-CoV-2 infection and expression.
Mycoplasma contamination	All the cell lines used tested negative for mycoplasma contamination.
Commonly misidentified lines<br>(See ICLAC register)	No commonly misidentified lines were used in this study.
